# Synthesis and characterization of cationic dicarbonyl Fe(II) PNP pincer complexes

**DOI:** 10.1007/s00706-016-1811-x

**Published:** 2016-08-06

**Authors:** Mathias Glatz, Christian Schröder-Holzhacker, Bernhard Bichler, Berthold Stöger, Kurt Mereiter, Luis F. Veiros, Karl Kirchner

**Affiliations:** 1 Institute of Applied Synthetic Chemistry, Vienna University of Technology, Getreidemarkt 9/163, 1060 Vienna, Austria; 2 Institute of Chemical Technologies and Analytics, Vienna University of Technology, Getreidemarkt 9, 1060 Vienna, Austria; 3 Centro de Química Estrutural, Instituto Superior Técnico, Universidade de Lisboa, Av. Rovisco Pais No. 1, 1049-001 Lisbon, Portugal

**Keywords:** Iron complexes, PNP pincer ligands, Carbon monoxide, DFT calculations

## Abstract

**Abstract:**

In the present work, we have prepared a series of octahedral Fe(II) complexes of the type *trans*-[Fe(PNP)(CO)_2_Cl]^+^—PNP are tridentate pincer-type ligands based on 2,6-diaminopyridine. These complexes are formed irrespective of the size of the substituents at the phosphorus sites and whether *cis*-[Fe(PNP)(Cl_2_)(CO)] or *trans*-[Fe(PNP)(Cl_2_)(CO)] are reacted with CO in the presence of 1 equiv of silver salts. X-ray structures of representative complexes are presented. Based on simple bonding considerations the selective formation of *trans*-dicarbonyl Fe(II) complexes is unexpected. In fact, DFT calculations confirm that *trans*-dicarbonyl complexes are indeed thermodynamically disfavored over the respective *cis*-dicarbonyl compounds, but are favored for kinetic reasons.

**Graphical abstract:**

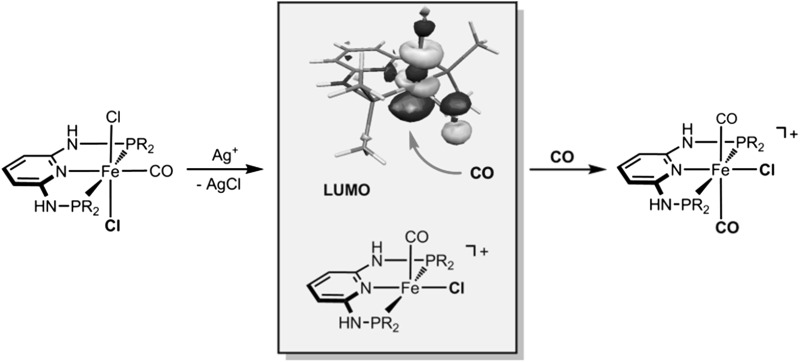

## Introduction

As part of our ongoing research on the synthesis and reactivity of iron(II) PNP pincer complexes [1–3], we recently prepared the cationic dicarbonyl complex *trans*-[Fe(PNP-*i*Pr)(CO)_2_Cl]^+^ (PNP-*i*Pr = *N,N*′-bis(diisopropyl)-2,6-diaminopyridine) (*trans*-**2a**) as shown in Scheme 1 [4]. The formation of this complex was somewhat unexpected as it features two CO ligands in a mutual *trans* position. In fact, simple bonding considerations suggest that the unobserved *cis* isomers are the more stable one. This was indeed also supported by DFT calculations. This complex is interesting, since the *trans* CO arrangement makes one of the CO ligands comparatively labile which can be replaced by other potential ligands. Accordingly, *trans*-[Fe(PNP-*i*Pr)(CO)_2_Cl]*X* with *X* = BF_4_^−^ turned out to be an efficient precatalyst for the coupling of aromatic aldehydes with ethyl diazoacetate to selectively give 3-hydroxyacrylates rather than β-keto esters [5].

**Figure:**
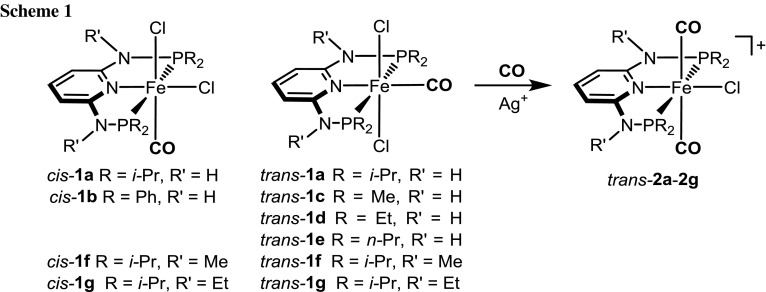

In continuation of our studies on iron PNP complexes, we herein report on the synthesis and reactivity of a series octahedral Fe(II) carbonyl complexes bearing both sterically little demanding as well as bulky PNP ligands in order to probe whether sterics influences the preference for a *trans*- over a *cis*-dicarbonyl arrangement. Moreover, we investigate the impact of the NR linker on the outcome of these reactions.

## Results and discussion

Treatment of complexes *cis*-**1b** and *trans*-**1c–1g** (**1f** and **1g** are mixtures of *cis* and *trans* isomers) with 1 equiv of Ag^+^ salts (with SbF_6_^−^, BF_4_^−^, or CF_3_SO_3_^−^ as counterions) in THF or acetone in the presence of CO at room temperature selectively afforded the cationic complexes *trans*-[Fe(κ^3^*P*,*N*,*P*-PNP)(CO)_2_*X*]^+^ (*trans*-**2b–2g**) in 78–98 % isolated yields (Scheme 1). The respective *cis*-dicarbonyl complexes were not observed and, hence, sterics and also the amine linker (NR) apparently do not influence the preference for a *trans*-dicarbonyl geometry. This is also supported by DFT calculations (vide infra). These complexes are thermally robust red solids that are air stable both in the solid state and in solution for several days. Characterization was accomplished by elemental analysis and ^1^H, ^13^C{^1^H}, ^31^P{^1^H} NMR and IR spectroscopy. In addition, the solid state structures of *trans*-**2b**, *trans*-**2d**, *trans*-**2f**, and *trans*-**2g** were determined by single-crystal X-ray diffraction.

In the IR spectrum, as expected, the CO ligands exhibit only one band between 1979 and 2031 cm^−1^ for the mutually *trans* CO ligands which are assigned to the asymmetric CO stretching frequency. The symmetric CO stretching band is IR inactive and not observed. The ^31^P{^1^H} NMR spectrum of complexes *trans***-2b–2g** show singlet resonances at 85.0, 92.3, 100.7, 96.7, 130.6, and 132.8 ppm, respectively. In the ^13^C{^1^H} NMR spectrum the two CO ligands exhibit a single low-intensity triplet resonance in the range of 207.2–211.8 ppm, thus clearly revealing that the two CO ligands are *trans* to one another.

**Fig. 1:**
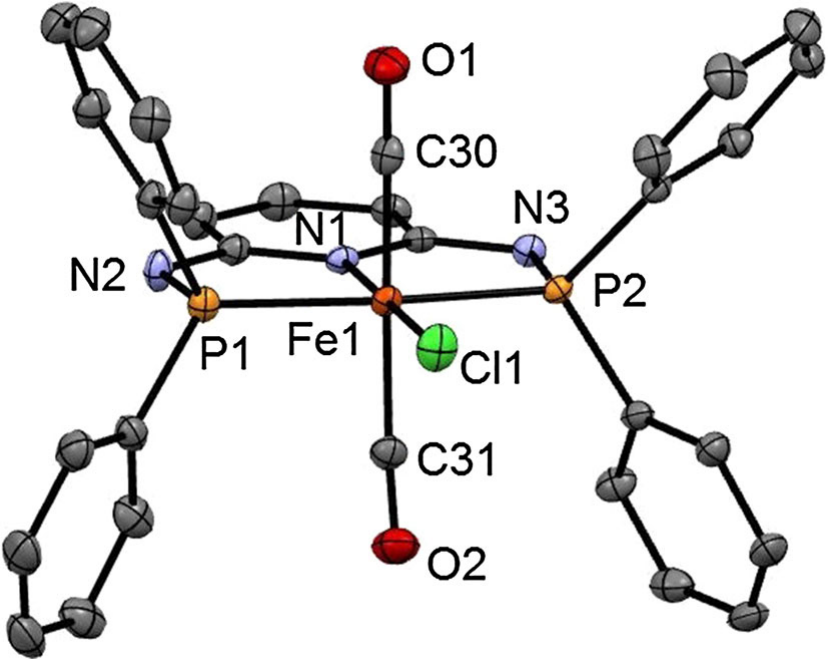
Structural view of *trans*-[Fe(PNP-Ph)(CO)_2_Cl]SbF_6_ (*trans*-**2a**) showing 50 % thermal ellipsoids (H atoms and counterion omitted for clarity). Selected bond lengths (Å) and bond angles (°): Fe1–Cl1 2.3029(7), Fe1–P2 2.2190(7), Fe1–P1 2.2317(7), Fe1–C30 1.824(3), Fe1–C31 1.850(3), Fe1–N1 1.977(2), P2–Fe1–P1 168.33(3), C30–Fe1–C31 172.6(1)

**Fig. 2:**
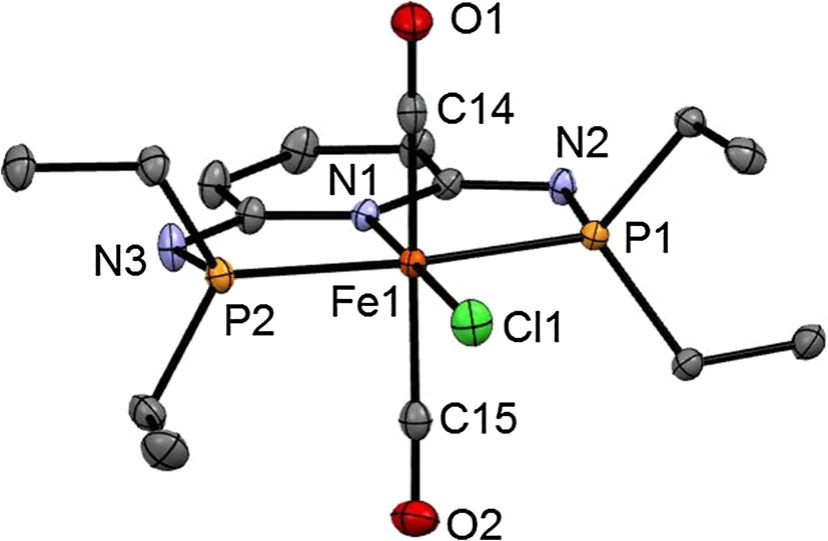
Structural view of *trans*-[Fe(PNP-Et)(CO)_2_Cl]CF_3_SO_3_ (*trans*-**2c**) showing 50 % thermal ellipsoids (H atoms and counterion omitted for clarity). Selected bond lengths (Å) and bond angles (°): Fe1–Cl1 2.3116(4), Fe1–P1 2.2265(4), Fe1–P2 2.2302(4), Fe1–N1 1.983(1), Fe1–C14 1.823(1), Fe1–C15 1.837(1), P1–Fe1–P2 167.82(2), C14–Fe1–C15 172.15(6)

**Fig. 3:**
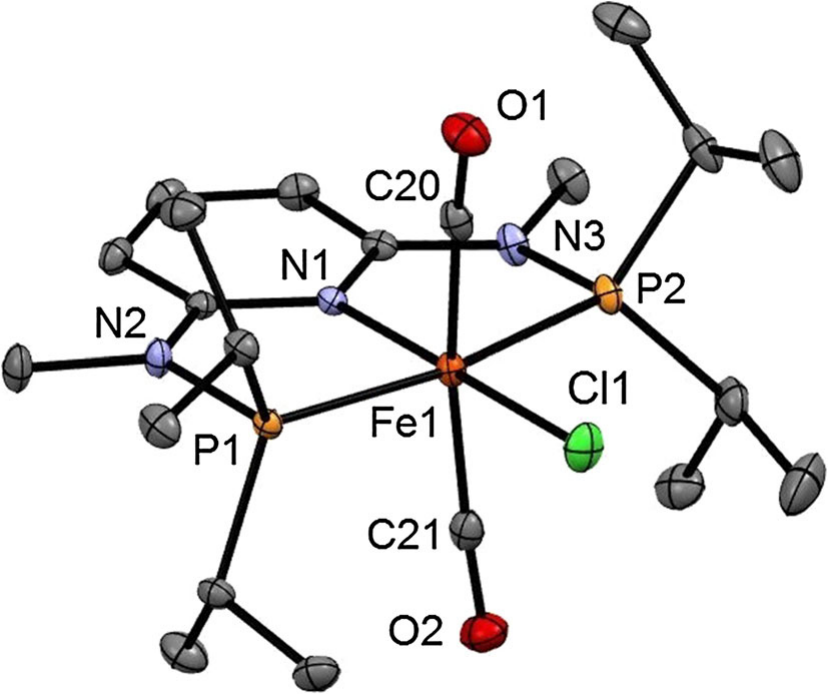
Structural view of *trans*-[Fe(PNP^Me^-*i*Pr)(CO)_2_Cl]BF_4_ (*trans*-**2e**) showing 50 % thermal ellipsoids (H atoms and counterion omitted for clarity). Selected bond lengths (Å) and bond angles (°): Fe1–Cl1 2.3009(5), Fe1–P1 2.2507(5), Fe1–P2 2.2455(5), Fe1–N1 1.976(1), Fe1–C20 1.818(1), Fe1–C21 1.819(1), P1–Fe1–P2 168.33(2), C20–Fe1–C21 168.71(7)

**Fig. 4:**
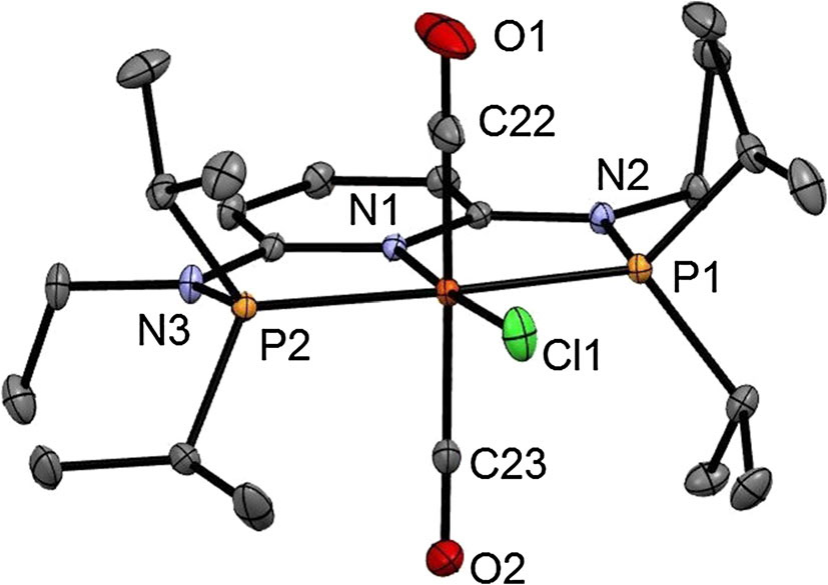
Structural view of *trans*-[Fe(PNP^Et^-*i*Pr)(CO)_2_Cl]BF_4_ (*trans*-**2f**) showing 50 % thermal ellipsoids (H atoms and counterion omitted for clarity). Selected bond lengths (Å) and bond angles (°): Fe1–Cl1 2.3034(3), Fe1–P1 2.2494(3), Fe1–P2 2.2598(3), Fe1–N1 1.9713(7), Fe1–C22 1.8126(10), Fe1–C23 1.8316(8), P1–Fe1–P2 169.14(1), C22–Fe1–C23 174.40(5)

Structural views of *trans*-**2b**, *trans*-**2d**, *trans*-**2f**, and *trans*-**2g** are depicted in Figs. 1, 2, 3 and 4 with selected bond distances and angles reported in the captions. All complexes adopt a distorted octahedral geometry around the metal center with the CO ligands in *trans* position to one another. The PNP ligand is coordinated to the iron center in a typical tridentate meridional mode, with P–Fe–P angles between 167.8° and 169.1°. The C_(CO)_–Fe–C_(CO)_ angles vary between 168.7° and 174.4°. The compounds with NH linkers show, as a typical feature, hydrogen bonds between the NH-groups of the cationic Fe(PNP) complexes and the counterions BF_4_^−^ and CF_3_SO_3_^−^.

**Fig. 5:**
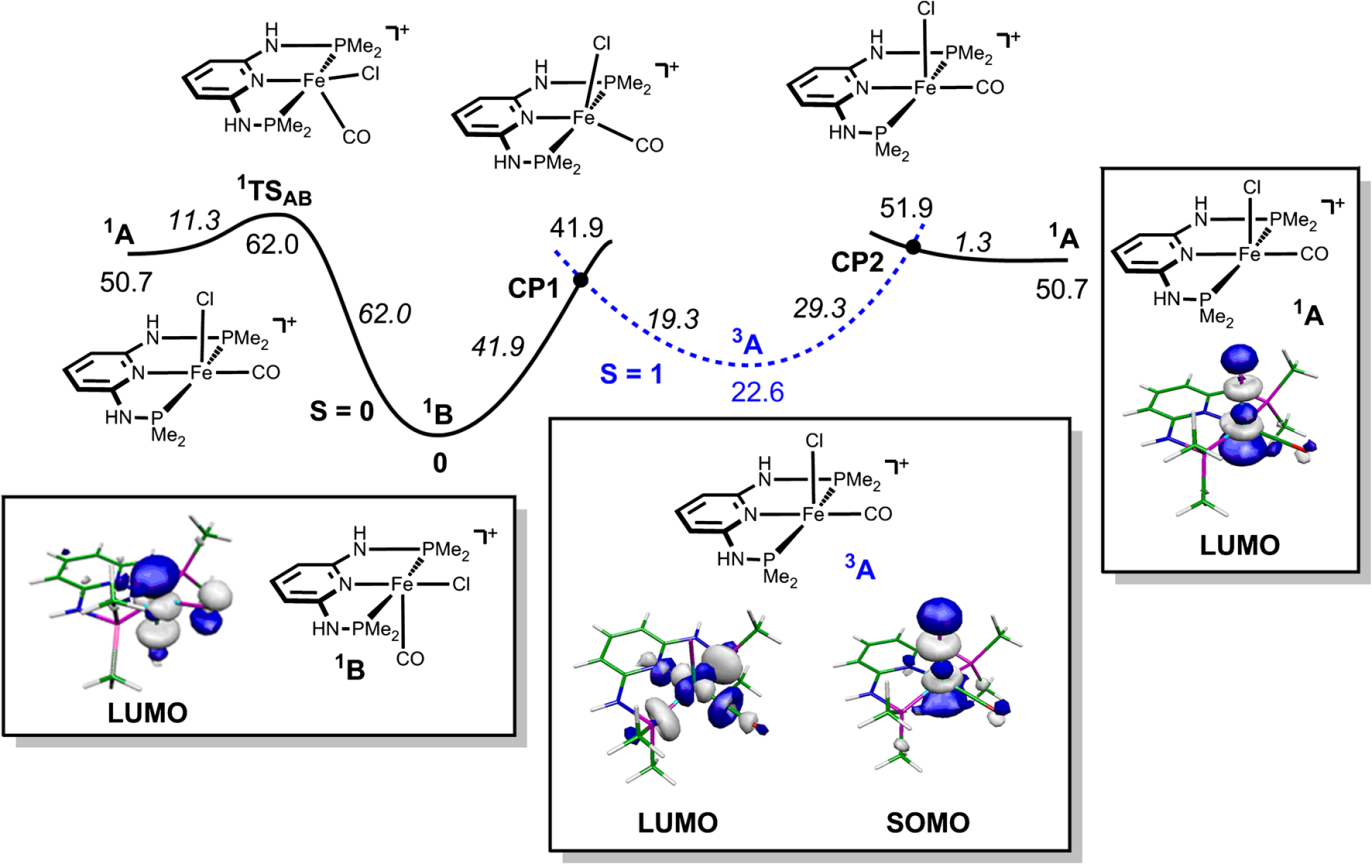
Energy profile (DFT/OPBE) for the *cis/trans* isomerization of pentacoordinated intermediates [Fe(PNP-Me)(CO)Cl]^+^ with the LUMO’s and the SOMO of ^1^ **A**, ^**1**^ **B**, and ^**3**^ **A**, respectively. The energy values (kJ mol^−1^) are referred to the cationic singlet intermediate [Fe(PNP-Me)(CO)Cl]^+^ (^**1**^ **B**). The *plain curve* corresponds to the spin singlet surface (*S* = 0) and the* dashed curve* corresponds to the spin triplet surface (*S* = 1)

To better understand why *trans*-dicarbonyl complexes are preferred over *cis*-dicarbonyl complexes, DFT calculations were performed with the *N*^2^,*N*^6^-bis(dimethylphosphanyl)-pyridine-2,6-diamine ligand (PNP-Me) as model. The starting point of our calculations are the coordinatively unsaturated cationic intermediates [Fe(PNP-Me)(CO)Cl]^+^ (**A** and/or **B**), which are formed from *trans*-[Fe(κ^3^*P*,*N*,*P*-PNP-Me)(CO)Cl_2_] (*trans*-**1c**) upon irreversible removal of chloride with silver salts (Scheme 2). The analogous *cis* isomer is experimentally not accessible. The energy profile (DFT/OPBE) for the *cis/trans* isomerization of [Fe(PNP-Me)(CO)Cl]^+^ is shown in Fig. 5.

**Figure:**
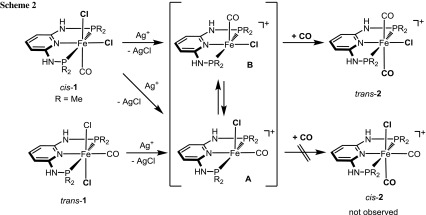

According to the calculations both cationic pentacoordinated intermediates **A** and **B** adopt a square pyramidal geometry where the Cl and the CO ligands, respectively, are in the apical position. The singlet ground state ^**1**^**B** is the energetically favored species by 22.6 and 50.7 kJ mol^−1^, respectively, over the singlet and triplet states of **A** (^**1**^**A**, ^**3**^**A**) (Fig. 5). In the case of **B**, no stable triplet state was found. **A** and **B** were found to interconvert readily via two pathways. ^**1**^**A** is able to isomerize along the spin singlet surface (*S* = 0) to give ^**1**^**B** with a small energy barrier of 11.3 kJ mol^−1^. This reaction proceeds via transition state ^**1**^**TS**_**AB**_. In the second pathway, ^**1**^**A** undergoes two consecutive spin state changes (spin crossover) from *S* = 0 to *S* = 1 and back to *S* = 0. The minimum energy crossing point^1^ between the potential energy surfaces of the two spin states *S* = 0 to *S* = 1 (**CP2**) is easily accessible lying merely 1.3 kJ mol^−1^ above ^**1**^**A**. The second spin state change from *S* = 1 to *S* = 0 proceeds via **CP1** with a barrier of 19.3 kJ mol^−1^.

Finally, the experimentally isolated *trans*-**2c** (which is actually is less stable than *cis*-**2c** by 17.2 kJ mol^−1^) is formed by an essentially barrierless addition of CO to ^**1**^**B** which is the most stable and predominant species lying 50.7 kJ mol^−1^ lower in energy than ^**1**^**A**. In general, CO addition at singlet intermediates is generally more favorable than at triplet intermediates as can be seen by examining the frontier orbitals of the relevant species. The LUMO of the pentacoordinated intermediates with a singlet spin state (^**1**^**A** and ^**1**^**B**) are formed mainly by *z*^2^-type orbitals centered at the Fe-atom and pointing towards the empty coordination position (Fig. 5). Therefore, these orbitals are ready to receive a pair of electrons from a ligand that occupies the sixth coordination site (CO in this case) and establish the corresponding σ-bond. In the case of spin triplet intermediate (^**3**^**A**), this orbital is occupied being, in fact, the highest single occupied molecular orbital (SOMO) of this species (Fig. 5). This is easily available to receive the electron pair from an incoming CO rendering addition of this ligand a difficult process. In fact, the first empty orbital (LUMO) in the case of the triplet intermediate corresponds to an *x*^2^–*y*^2^-type orbital which is centered on the metal and is antibonding (σ^*^) with respect to the four ligands in the equatorial plane.

## Conclusion

In the present work we have prepared, spectroscopically and structurally characterized several octahedral iron(II) complexes of the type *trans*-[Fe(PNP)(CO)_2_*X*]^+^. These complexes are formed irrespective of the size of the substituents at the phosphorus sites and whether *cis*-[Fe(PNP)(Cl_2_)(CO)] or *trans*-[Fe(PNP)(Cl_2_)(CO)] are reacted with CO in the presence of 1 equiv of silver salts. Based on simple bonding considerations the selective formation of *trans*-dicarbonyl Fe(II) complexes is unexpected. DFT calculations indeed confirm that *trans*-dicarbonyl complexes are thermodynamically disfavored over the respective *cis*-dicarbonyl compounds. The key to an understanding of this unexpected selectivity is the fact that upon irreversible removal of a chloride ligand from [Fe(PNP)(CO)Cl_2_] pentacoordinate intermediates [Fe(PNP)(CO)Cl]^+^ of two conformations, one with the chloride in the apical and CO in the basal position (**A**) and vice versa (**B**), are formed. The subsequent carbonylation process depends strongly on the complex geometry of the 16e intermediates [Fe(PNP)(CO)Cl]^+^, i.e., **A** vs. **B**, which in turn determines the spin state (*S* = 0 or *S* = 1) and consequently the reactivity and also the stability of these intermediates. According to calculations, **B** in the singlet ground state is the most stable and also kinetically the most accessible intermediate in solution. The formation of *trans*-[Fe(PNP)(CO)_2_Cl]^+^ is kinetically controlled with ^**1**^**B** being the key intermediate. The mechanism deduced from DFT calculations is in full agreement with experimental findings.

## Experimental

All manipulations were performed under an inert atmosphere of argon by using Schlenk techniques or in an MBraun inert-gas glovebox. The solvents were purified according to standard procedures [7]. The deuterated solvents were purchased from Aldrich and dried over 4 Å molecular sieves. Complexes *cis*-[Fe(κ^3^*P*,*N*,*P*-PNP-Ph)(CO)Cl_2_] (*cis*-**1b**), *trans*-[Fe(κ^3^*P*,*N*,*P*-PNP-Me)(CO)Cl_2_] (*trans*-**1c**), *trans*-[Fe(κ^3^*P*,*N*,*P*-PNP-Et)(CO)Cl_2_] (*trans*-**1d**), *trans*-[Fe(κ^3^*P*,*N*,*P*-PNP-*n*Pr)(CO)Cl_2_] (*trans*-**1e**), *cis*/*trans*-[Fe(PNP^Me^-*i*Pr)(CO)Cl_2_] (*cis*/*trans*-**1f**), and *cis*/*trans*-[Fe(PNP^Et^-*i*Pr)(CO)Cl_2_] (*cis*/*trans*-**1** **g**) were prepared according to the literature [8]. ^1^H, ^13^C{^1^H}, and ^31^P{^1^H} NMR spectra were recorded on Bruker AVANCE-250 and AVANCE-400 spectrometers. ^1^H and ^13^C{^1^H} NMR spectra were referenced internally to residual protio-solvent and solvent resonances, respectively, and are reported relative to tetramethylsilane (*δ* = 0 ppm). ^31^P{^1^H} NMR spectra were referenced externally to H_3_PO_4_ (85 %) (*δ* = 0 ppm).

### *Trans*-[(chloro)[*N*^2^,*N*^6^-bis(diphenylphosphanyl)pyridine-2,6-diamine](dicarbonyl)iron(II)] tetrafluoroborate (*trans*-[Fe(κ^3^*P*,*N*,*P*-PNP-Ph)(CO)_2_Cl]BF_4_) (*trans*-**2b**, C_31_H_25_BClF_4_FeN_3_O_2_P_2_)

Complex *cis*-**1b** (200 mg, 0.316 mmol) was dissolved in 10 cm^3^ THF, CO gas was bubbled through the solution and 62 mg AgBF_4_ (0.316 mmol) was added. After 4 h the red solution was filtered over Celite and the solvent was evaporated. The red powder was washed with 20 cm^3^ Et_2_O and dried under vacuum. Yield 180 mg (85 %); ^1^H NMR (acetone-*d*_*6*_, 20 °C): *δ* = 9.50 (s, 2H, NH), 8.10 (m, 5H, Ph, py^4^), 7.71 (m, 18H, Ph, py^3,5^) ppm; ^13^C{^1^H} NMR (CD_2_Cl_2_): *δ* = 207.2 (t, *J* = 25.8 Hz, CO), 161.3 (py), 141.8 (py), 134.6–133.2 (Ph), 132.10 (Ph), 131.0–129.8 (Ph), 129.2 (t, *J* = 5.4 Hz, Ph), 102.2 (py) ppm; ^31^P{^1^H} NMR (acetone-*d*_*6*_, 20 °C): *δ* = 85.0 ppm; IR (ATR, 20 °C): $\bar{\nu }$ = 2031 (*ν*_C=O_) cm^−1^.

### *Trans*-[(chloro)[*N*^2^,*N*^6^-bis(dimethylphosphanyl)pyridine-2,6-diamine](dicarbonyl)iron(II)] trifluoromethanesulfonate (*trans*-[Fe(κ^3^*P,N,P*-PNP-Me)(CO)_2_Cl]CF_3_SO_3_) (*trans*-**2c**, C_12_H_17_ClF_3_FeN_3_O_5_P_2_S)

CO was bubbled through a suspension of 100 mg *trans*-**1b** (0.26 mmol) and 67 mg AgCF_3_SO_3_ (0.26 mmol) in 7 cm^3^ acetone. The orange solution was then filtrated over Celite, evaporated to dryness and the obtained solid was washed with 10 cm^3^*n*-hexane. The orange powder was dried under reduced pressure. Yield 134 mg (98 %); ^1^H NMR (acetone-*d*_*6*_, 20 °C): *δ* = 8.46 (s, 2H, NH), 7.33 (t, *J*_*HH*_ = 7.9 Hz, 1H, py^4^), 6.23 (d, *J*_*HH*_ = 8.0 Hz, 2H, py^3,5^), 2.38 (m, 12H, CH_3_) ppm; ^13^C{^1^H} NMR (acetone-*d*_*6*_, 20 °C): *δ* = 210.3 (t, *J*_*CP*_ = 26.8 Hz, CO), 162.4 (t, *J*_*CP*_ = 7.5 Hz, py), 141.9 (py), 101.1 (t, *J*_*CP*_ = 3.8 Hz, py), 18.9 (t, *J*_*CP*_ = 17.2 Hz, CH_3_) ppm; ^31^P{^1^H} NMR (acetone-*d*_*6*_, 20 °C): *δ* = 92.3 ppm; IR (ATR): $\bar{\nu }$ = 1979 (*ν*_CO_) cm^−1^.

### *Trans*-[(chloro)[*N*^2^,*N*^6^-bis(diethylphosphanyl)pyridine-2,6-diamine](dicarbonyl)iron(II)] trifluoromethanesulfonate (*trans*-[Fe(κ^3^*P,N,P*-PNP-Et)(CO)_2_Cl]CF_3_SO_3_) (*trans*-**2c**, C_16_H_25_ClF_3_FeN_3_O_5_P_2_S)

This compound was prepared analogously to *trans*-**2b** with 120 mg *trans*-**1c** (0.27 mmol) and 70 mg AgCF_3_SO_3_ (0.27 mmol) as starting materials. The orange product was dried under reduced pressure. Yield: 153 mg (97 %). Crystals were grown from an acetone solution of **2c** by slow diffusion of Et_2_O. ^1^H NMR (acetone-*d*_*6*_, 20 °C): *δ* = 8.49 (2H, NH), 7.63 (1H, py^4^), 6.31 (d, *J*_*HH*_ = 5.2 Hz, 2H, py^3,5^), 2.90 (4H, CH_2_), 2.78 (4H, CH_2_), 1.51 (12H, CH_3_) ppm; ^13^C{^1^H} NMR (acetone-*d*_*6*_, 20 °C): *δ* = 210.5 (t, *J*_*CP*_ = 25.2 Hz, CO), 161.8 (t, *J*_*CP*_ = 6.9 Hz, py), 141.1 (py), 100.3 (py), 23.4 (t, *J*_*CP*_ = 15.3 Hz, CH_2_), 6.4 (CH_3_) ppm; ^31^P{^1^H} NMR (acetone-*d*_*6*_, 20 °C): *δ* = 100.7 ppm; IR (ATR): $\bar{\nu }$ = 2008 (*ν*_CO_) cm^−1^.

### *Trans*-[(chloro)[*N*^2^,*N*^6^-bis(dipropylphosphanyl)pyridine-2,6-diamine](dicarbonyl)iron(II)] trifluoromethanesulfonate (*trans*-[Fe(κ^3^*P,N,P*-PNP-nPr)(CO)_2_Cl]CF_3_SO_3_) (*trans*-**2d**, C_20_H_33_ClF_3_FeN_3_O_5_P_2_S)

This compound was prepared analogously to *trans*-**2b** using 150 mg *trans*-**1d** (0.30 mmol) and 78 mg AgCF_3_SO_3_ (0.30 mmol) as starting materials. The red–orange product was dried under reduced pressure. Yield: 177 mg (92 %); ^1^H NMR (acetone-*d*_*6*_, 20 °C): *δ* = 8.41 (2H, NH), 7.47 (t, *J*_*HH*_ = 7.9 Hz, 1H, py^4^), 6.41 (d, *J*_*HH*_ = 7.6 Hz, 2H, py^2,6^), 2.01 (m, 8H, CH_2_), 1.58 (m, 8H, CH_2_), 1.12 (t, *J*_*HH*_ = 7.1 Hz, 12H, CH_3_) ppm; ^13^C{^1^H} NMR (acetone-*d*_*6*_, 20 °C): *δ* = 210.4 (t, *J*_*CP*_ = 25.6 Hz, CO), 161.7 (t, *J*_*CP*_ = 6.8 Hz, py), 140.9 (py), 100.2 (t, *J*_*CP*_ = 3.7 Hz, py), 32.8 (t, *J*_*CP*_ = 14.3 Hz, CH_2_), 16.2 (CH_3_), 15.0 (t, *J*_*CP*_ = 7.8 Hz, CH_2_) ppm; ^31^P{^1^H} NMR (acetone-*d*_*6*_, 20 °C): *δ* = 96.7 ppm; IR (ATR): $\bar{\nu }$ = 2011 (*ν*_CO_) cm^−1^.

### *Trans*-[(chloro)[*N*^2^,*N*^6^-bis(diisopropylphosphanyl)-*N*^2^,*N*^6^-dimethylpyridine-2,6-diamine](dicarbonyl)iron(II)] tetrafluoroborate (*trans*-[Fe(κ^3^*P,N,P*-PNP^Me^-iPr)(CO)_2_Cl]BF_4_) (*trans*-**2e**, C_21_H_37_BClF_4_FeN_3_O_2_P_2_)

CO was bubbled through a solution of 150 mg *cis/trans*-**1e** (0.30 mmol) and 59 mg AgBF_4_ (0.30 mmol) in 15 cm^3^ of THF. The pink solution was stirred under CO atmosphere for 1 h; then the solvent was removed under reduced pressure. The residue was redissolved in 15 cm^3^ of CH_2_Cl_2_, filtered and the volume of the solvent was reduced to about 0.5 cm^3^. The product was precipitated by addition of 40 cm^3^ of pentane, collected on a glass frit, washed with 15 cm^3^ of *n*-pentane, and dried under vacuum. Yield: 141 mg (78 %); ^1^H NMR (CD_2_Cl_2_, 20 °C): *δ* = 7.53 (t, ^*3*^*J*_*HH*_ = 8.1 Hz, 1H, py^4^), 6.14 (d, ^*3*^*J*_*HH*_ = 8.2 Hz, 2H, py^3,5^), 3.19 (m, 4H, C*H*(CH_3_)_2_), 3.08 (s, 6H, NCH_3_), 1.53–1.42 (m, 24H, CH(C*H*_*3*_)_2_) ppm; ^13^C{^1^H} NMR (CD_2_Cl_2_, 20 °C): *δ* = 211.6 (t, ^*2*^*J*_*CP*_ = 24.7 Hz, CO), 163.0 (vt, ^*2*^*J*_*CP*_ = 7.4 Hz, py^2,6^), 142.2 (s, py^4^), 100.2 (vt, ^*3*^*J*_*CP*_ = 2.7 Hz, py^3,5^), 35.4 (s, NCH_3_), 32.0 (vt, ^*1*^*J*_*CP*_ = 11.2 Hz, *C*H(CH_3_)_2_), 18.5 (s, CH(*C*H_3_)_2_), 17.7 (s, CH(*C*H_3_)_2_) ppm; ^31^P{^1^H} NMR (CD_2_Cl_2_, 20 °C): *δ* = 130.6 ppm; IR (ATR): $\bar{\nu }$ = 2002 (*ν*_C=O_) cm^−1^.

### *Trans*-[(chloro)[*N*^2^,*N*^6^-bis(diisopropylphosphanyl)-*N*^2^,*N*^6^-diethylpyridine-2,6-diamine](dicarbonyl)iron(II)] tetrafluoroborate (*trans*-[Fe(κ^3^*P,N,P*-PNP^Et^-iPr)(CO)_2_Cl]BF_4_) (*trans*-**2f**, C_23_H_41_BClF_4_FeN_3_O_2_P_2_)

This complex was prepared analogously to *trans*-**2e** with 150 mg *cis/trans*-**1f** (0.29 mmol) and 56 mg AgBF_4_ (0.29 mmol) as starting materials. Yield: 131 mg (75 %); ^1^H NMR (CD_2_Cl_2_, 20 °C): *δ* = 7.54 (t, ^*3*^*J*_*HH*_ = 8.2 Hz, 1H, py^4^), 6.17 (d, ^*3*^*J*_*HH*_ = 8.2 Hz, 2H, py^3,5^), 3.58 (m, 4H, NC*H*_*2*_CH_3_), 3.18 (m, C*H*(CH_3_)_2_), 1.49–1.10 (m, 30H, NCH_2_C*H*_*3*_, CH(C*H*_*3*_)_2_) ppm; ^13^C{^1^H} NMR (CD_2_Cl_2_, 20 °C): *δ* = 211.8 (t, ^*2*^*J*_*CP*_ = 24.8 Hz, *C*O), 162.3 (vt, ^*2*^*J*_*CP*_ = 6.9 Hz, py^2,6^), 142.4 (s, py^4^), 101.2 (vt, ^*3*^*J*_*CP*_ = 2.6 Hz, py^3,5^), 43.3 (s, N*C*H_2_CH_3_), 31.4 (vt, ^*1*^*J*_*CP*_ = 10.8 Hz, *C*H(CH_3_)_2_), 19.1 (s, CH(*C*H_3_)_2_), 17.8 (s, CH(*C*H_3_)_2_), 13.0 (s, NCH_2_*C*H_3_) ppm; ^31^P{^1^H} NMR (CD_2_Cl_2_, 20 °C): *δ* = 132.8 ppm; IR (ATR): $\bar{\nu }$ = 2005 (*ν*_C=O_) cm^−1^.

### X-ray structure determination

X-ray diffraction data of *trans*-**2a**, *trans*-**2c**, *trans*-**2e**, and *trans*-**2f** (CCDC entries 1015363 (*trans*-**2a**), 1469956 (*trans*-**2c**), 1469957 (*trans*-**2e**), 1469958 (*trans***-2f**),) were collected at *T* = 100 K in a dry stream of nitrogen on Bruker Kappa APEX II diffractometer systems using graphite-monochromatized Mo-*K*α radiation (*λ* = 0.71073 Å) and fine sliced φ- and ω**-**scans. Data were reduced to intensity values with SAINT and an absorption correction was applied with the multi-scan approach implemented in SADABS [9]. The structures of *trans*-**2c**, *trans*-**2e**, and *trans*-**2f** were solved by charge flipping using SUPERFLIP [10] and refined against with JANA2006 [11]. The structure of *trans*-**2a** was solved with direct methods and refined against *F*2 with the SHELX software package [12]. Non-hydrogen atoms were refined anisotropically. The H atoms connected to C atoms were placed in calculated positions and thereafter refined as riding on the parent atoms. The H atoms of the amine functionalities were located in difference Fourier maps and freely refined. Molecular graphics were generated with the program MERCURY [13].

### Computational details

Calculations were performed using the Gaussian 09 software package, and the OPBE functional without symmetry constraints as already described previously [14].

## Footnotes

1 In the MECP both the energy as well as the geometry of the molecule are the same in the two spin states surfaces. Once that point (MECP) is reached, following the reaction coordinate, there is a given probability for the system to change spin state and hop from one PES to the other, giving rise to the “spin-forbidden” reaction. For more information about MECP and the kinetics of spin-forbidden reactions see for example Ref. [6].
